# Corneal nerve regeneration and ocular surface restoration following treatment with autologous serum eye drops in patients with paralytic lagophthalmos and neurotrophic keratitis

**DOI:** 10.1007/s00417-026-07388-w

**Published:** 2026-07-17

**Authors:** Luca Lucchino, Elvia Mastrogiuseppe, Francesca Giovannetti, Mattia D’Andrea, Giulia Mecarelli, Alice Bruscolini, Alessandro Lambiase, Marco Marenco

**Affiliations:** Department of Sense Organs, Sapienza – University of Rome, Viale del Policlinico 155, Rome, 00161 Italy

**Keywords:** Autologous serum eye drops (ASED), Neurotrophic keratitis (NK), Paralytic lagophthalmos (PL), In vivo confocal microscopy (IVCM), Corneal nerve regeneration

## Abstract

**Purpose:**

To investigate the effect of 20% autologous serum eye drops (ASED) in improving ocular surface condition and promoting corneal nerve regeneration in patients with Paralytic Lagophthalmos (PL) and Neurotrophic Keratitis (NK).

**Methods:**

This prospective study included 11 patients with unilateral NK and PL treated with 20% ASED for 2 months. Ocular surface assessment included Schirmer I test, non-invasive tear film break-up time (NIBUT), Oxford staining score, Ocular Surface Disease Index (OSDI) and corneal esthesiometry. In vivo confocal microscopy (IVCM) was used to quantify changes in corneal subbasal nerve plexus, evaluating corneal nerve fiber length (CNFL), nerve fiber density (CNFD), and nerve branch density (CNBD). Pre- and post-treatment values were compared.

**Results:**

Ten patients completed the study. ASED treatment significantly improved Schirmer I values (mean: 10.3 ± 4.7 mm to 19.7 ± 12.1 mm; *p* = 0.041), OSDI scores (21.1 ± 12.9 to 17.0 ± 10.0; *p* = 0.009), and corneal sensitivity readings (central esthesiometry: 3.4 ± 1.9 mm to 5.0 ± 1.2 mm; *p* = 0.005). IVCM revealed significant increases in CNFL (11.1 ± 6.4 to 14.7 ± 7.3 mm/mm²; *p* = 0.006) and CNFD (23.1 ± 13.2 to 33.8 ± 11.9 fibers/mm²; *p* = 0.012). One patient discontinued treatment due to ocular discomfort.

**Conclusion:**

Topical 20% ASED improved both functional and structural parameters in patients with PL and NK, suggesting neuroregenerative potential. These findings support the use of ASED as a therapeutic option in complex ocular surface disease, with IVCM as a valuable tool for monitoring nerve recovery.

**Supplementary Information:**

The online version contains supplementary material available at https://doi.org/10.1007/s00417-026-07388-w.

## Key messages

*****What is known***:**

- Autologous serum eye drops (ASED) are widely used to treat ocular surface disorders and are known to support epithelial healing in neurotrophic keratitis (NK).

*****What is new***:**

- Treatment with 20% autologous serum eye drops (ASED) can promote both structural and functional regeneration of the corneal subbasal nerve plexus in patients with paralytic lagophthalmos (PL) and neurotrophic keratitis (NK), as highlighted by in vivo confocal microscopy.
- Significant improvements were observed in corneal sensitivity, tear production, and ocular surface health, supporting the neurotrophic and restorative potential of ASED in clinically challenging patient population.

**Supplementary Information:**

The online version contains supplementary material available at https://doi.org/10.1007/s00417-026-07388-w.

## Introduction

Paralytic lagophthalmos (PL), the most common form of lagophthalmos, arises from peripheral facial nerve (VII) paralysis, leading to impaired orbicularis oculi function and incomplete eyelid closure. Etiologies include trauma, infections, tumor infiltration, iatrogenic injury, metabolic disorders, and congenital or neurological conditions [1, 2]. 

The cornea is the most densely innervated tissue in the human body. Innervation is supplied by long ciliary nerves originating from the ophthalmic branch of the trigeminal nerve. These fibers form a stromal plexus, lose their myelination, and terminate in the epithelium as the corneal subbasal nerve plexus (CSNP) [3]. Corneal innervation is essential for maintaining epithelial integrity, proliferation, and wound healing. Trigeminal nerve damage leads to reduced or absent corneal sensitivity and may result in neurotrophic keratitis (NK) [4]. Clinical manifestations range from punctate keratopathy to ulcers, melting, perforation, stromal scarring, neovascularization, tear film instability, and reduced blink reflex. Lesions can occur at various levels—from the brainstem to peripheral branches—and may be caused by viral infections (e.g., herpes simplex), trauma, systemic diseases such as diabetes or multiple sclerosis, as well as intracranial tumors, aneurysms, or neurosurgical procedures [5]. 

Inadequate eyelid closure due to PL, combined with reduced corneal sensitivity from corneal nerve damage, can lead to severe keratopathy, which may progress to corneal ulceration, perforation, and permanent vision loss [6]. Management typically includes non-preserved artificial tears and ophthalmic ointments to improve tear film stability and protect the ocular surface. Temporary measures such as lateral tarsorrhaphy, botulinum toxin-induced ptosis, or removable eyelid weights may also be employed, particularly when facial nerve recovery is anticipated [7, 8]. Bandage contact lenses are often unsuitable in this setting due to localized drying of the lens surface, which can cause mechanical abrasions, poor fitting, and spontaneous lens loss [9]. 

Autologous serum eye drops (ASED) are widely used in ophthalmology to treat various ocular surface disorders, including severe dry eye, graft-versus-host disease, recurrent or persistent corneal erosions, and NK [10]. Rich in bioactive components, autologous serum provides essential factors that support proliferation, migration, and differentiation of ocular surface epithelial cells. These include epidermal growth factor (EGF), transforming growth factor β (TGF-β), vitamin A, fibronectin, albumin, α-2 macroglobulin, and neuropeptides such as substance P and insulin-like growth factor 1. Furthermore, several of these growth factors exhibit neurotrophic properties and have been shown to promote nerve regeneration on the ocular surface [11]. 

In vivo confocal microscopy (IVCM) has recently emerged as a valuable non-invasive technique for high-resolution imaging of the cornea, offering detailed insights into its cellular and microstructural organization in both healthy and diseased states [12]. By enabling direct visualization of corneal nerves, IVCM serves as a key diagnostic tool to assess nerve damage and monitor regeneration following treatment [13]. 

The aim of this study is to evaluate the effects of ASED on the ocular surface and CSNP in patients with PL and NK.

## Materials and methods

### Study design

This single-center, prospective study assessed the effect of topical 20% ASED in patients with PL and NK. The study was conducted at the Department of Sense Organs, Sapienza University of Rome, Italy, after approval by the Ethics Committee of Sapienza University of Rome (Prot. N. 0787/2021). The research adhered to the Tenets of the Declaration of Helsinki, and informed consent was obtained from all participants prior to enrolment.

### Clinical cohort

Patients with PL were referred from the Maxillofacial Surgery and Neurosurgery departments to the Rare, Inflammatory, and Degenerative Diseases Unit of the Department of Sense Organs, Sapienza University of Rome. All patients underwent ophthalmologic evaluation, and those with a confirmed diagnosis of concomitant NK were screened for eligibility and enrolled after providing informed consent.

#### Inclusion criteria were as follows

patients aged ≥ 18 years; lagophthalmos ≥ 5 mm secondary to peripheral facial nerve paralysis persisting for at least six months [14]; presence of keratopathy (Oxford score > 0.5) unresponsive to tear substitutes or unsuitable for therapeutic contact lenses due to poor fitting or mechanical complications; OSDI (Ocular Surface Disease Index) score > 10; diagnosis of unilateral stage 1 NK following neurosurgery, according to the Mackie classification [5, 15]. The fellow eye showed normal corneal sensitivity and no epithelial staining.

#### Exclusion criteria included

use of topical corticosteroids or immunosuppressants within the past 30 days; contact lens wear; history of previous corneal surgery or preexisting corneal diseases; ocular surgery within the past six months; current or previous glaucoma treatment; active infection or inflammation; diabetes mellitus; history of peripheral neuropathy; or inability to follow instructions. Patients with positive serologic screening for hepatitis B or C, syphilis, or HIV were excluded from the study.

### Clinical assessment and measurements

Baseline assessments included patient demographics, ocular history, and comorbidities. Comprehensive ophthalmologic examination included OSDI questionnaire; Schirmer I test; non-invasive tear film break-up time (NIBUT) using a corneal Sirius topographer (CSO, Florence, Italy) [16]; dynamic measurement of lagophthalmos degree using a ruler; visual acuity testing; Cochet-Bonnet esthesiometry; slit-lamp examination with fluorescein staining graded using the Oxford Grading System; and in vivo confocal microscopy (IVCM) for corneal nerve fiber length (CNFL), nerve fiber density (CNFD), and nerve branch density (CNBD). The same measurements were repeated 60 days ± 2 days at the end of the treatment period.

Corneal sensitivity was measured using the Cochet-Bonnet esthesiometer (Luneau Ophthalmologie, Chartres, France), which uses a 0.12 mm diameter nylon monofilament ranging from 5 to 60 mm in length. The filament was applied perpendicularly to the central cornea and four quadrants. If no blink reflex occurred, the filament was shortened by 1.0 cm increments until a positive response was elicited. The threshold was confirmed by extending the filament by 0.5 cm. The longest filament length eliciting a positive response was recorded [6]. All measurements were performed under standardized environmental conditions (humidity 50–70%, temperature 18–25 °C). The filament was sterilized and retracted after each use.

### IVCM procedure and analysis

Laser IVCM (HRT3-RCM; Heidelberg Retina Tomograph 3 with the Rostock Cornea Module, Heidelberg, Germany) was used to examine the central cornea [17]. The device uses a 670 nm red diode laser and a 60x water-immersion objective lens (NA 0.9), achieving up to 800× magnification. Images (400 × 400 μm field size; 2 μm resolution; 4 μm optical section thickness) were acquired in sequence mode (100 images in 10 s). The corneal surface was anesthetized with oxybuprocaine hydrochloride 0.4%, and polyacrylic acid gel (Viscotears) was applied as a coupling agent. Participants were instructed to fixate on a target to ensure imaging of the central cornea. Full-thickness scans were obtained through 4–6 sequences, yielding 50–100 images of the CSNP. Three representative images were selected by a masked observer based on focus, contrast, and absence of artifacts. CNFL (mm/mm²), CNFD (fibers/mm²), and CNBD (branches/mm²) were analyzed using NeuronJ (http://www.imagescience.org/meijering/software/neuronj/) a semi-automated tracing software for ImageJ [17, 18]. Inter- and intra-observer variability was assessed, with discrepancies resolved by a third observer.

### Preparation of autologous serum eye drops and treatment protocol

ASED were prepared following standardized protocols [19]. Patients were screened for hepatitis B and C, syphilis, and HIV (HbsAg, HCV antibodies, HIV I/II, NAT). Only seronegative individuals were eligible. A total of 100 ml of peripheral blood was collected under aseptic conditions using sterile vacuum tubes without anticoagulants. After two hours at room temperature for clotting, samples were centrifuged at 4200 RPM for six minutes. The supernatant was filtered (0.2 μm pore size), diluted 1:4 with balanced salt solution (BSS) to obtain a 20% concentration, and aliquoted into sterile 2 ml ophthalmic containers. Aliquots were stored at − 20 °C; the in-use bottle was refrigerated at + 4 °C and discarded after 24 h. Patients were instructed to instill ASED six times daily for two months in the affected eye, and to discontinue different topical treatments including artificial tears, lubricating gels, or ointments, throughout the entire study period. No additional surgical or medical treatments for PL were initiated or modified during the study period. A follow-up examination was performed after two weeks to assess adverse events. At the end of treatment, baseline assessments were repeated by the same examiner.

### Statistical analysis

Descriptive statistics, including medians and means with standard deviations, were calculated. Distribution was assessed using the Shapiro-Wilk test. Pre- and post-treatment (V0 and V1) values were compared using the Wilcoxon signed-rank test or paired t-test, depending on normality. Correlations between clinical and structural changes were assessed using Pearson or Spearman coefficients. Analyses were performed using GraphPad Prism (v8.5.1, GraphPad Software, San Diego, CA, USA) and JASP (v0.17.1). Statistical significance was set at *p* < 0.05.

## Results

### Clinical findings

A total of 11 eyes from 11 patients (6 females, 5 males; mean age: 60.0 ± 6.1 years) were included in the study. Eight patients (72.7%) had iatrogenic facial nerve paralysis following surgical removal of neoplasms (benign or malignant), and three patients (27.3%) had paralysis secondary to tumor infiltration of the facial nerve. In all cases, lagophthalmos was secondary to peripheral facial nerve paralysis, persisting for an average of 5.5 ± 5.2 years (range: 2–20 years). At the time of enrolment, no patient was using topical medications other than hyaluronic acid tear substitutes (0.2–0.4%). Additionally, one patient was practicing nocturnal occlusion with Steri-Strips. Three patients had undergone previous surgical treatments for lagophthalmos, including implantation of weighted gold in the upper eyelid (*n* = 2) or fat (*n* = 1). Baseline slit-lamp examination was consistent with stage 1 NK (Mackie classification) characterized by predominantly central superficial punctate keratopathyand mild epithelial irregularity. Bell’s phenomenon was preserved. The characteristics of the study population are summarized in Table 1.

**Table 1:** Baseline demographic characteristics of the study population

ID	Sex	Age (years)	Eye	BCVA (logMAR)	Etiology of paralysis	Paralysis onset (years)	Previous treatments
1	F	61	RE	0,05	Iatrogenic, vestibular schwannoma	Since 2021	tear substitutes
2	M	59	LE	0,40	Neoplastic, parotid tumor	Since 2022	tear substitutes
3	M	64	LE	0,10	Iatrogenic, vestibular schwannoma	Since 2022	tear substitutes + nocturnal occlusion with Steri-Strips
4	F	72	RE	0,15	Iatrogenic, meningioma	Since 2018	tear substitutes + autologous fat graft
5	F	52	RE	0,30	Iatrogenic, vestibular schwannoma	Since 2016	tear substitutes + implantation of weighted gold
6	F	48	LE	0,00	Iatrogenic, vestibular schwannoma	Since 2017	tear substitutes
7	F	63	RE	0,10	Iatrogenic, meningioma	Since 2017	tear substitutes
8	M	56	LE	0,22	Neoplastic, parotid tumor	Since 2017	tear substitutes
9	M	65	RE	0,00	Iatrogenic, vestibular schwannoma	Since 2008	tear substitutes + implantation of weighted gold
10	F	55	RE	0,40	Neoplastic, parotid tumor	Since 2002	tear substitutes
11	M	61	RE	0,05	Iatrogenic, vestibular schwannoma	Since 2021	tear substitutes

*BCVA* best corrected visual acuity, *RE *right eye, *LE* left eye

The patients were reviewed after two weeks of treatment. One patient reported ocular discomfort; therefore, he was advised to discontinue the treatment and dropped out of the study. The remaining ten patients continued enrolment in the study and were included in the final analysis.

After 2 months of treatment with 20% ASED, statistically significant improvements were observed in tear production and corneal sensitivity. The Schirmer I test analysis demonstrated scores rising from 10.3 ± 4.7 mm pre-treatment to 19.7 ± 12.1 mm post-treatment (*p* = 0.041) (Table 2). Corneal sensitivity, assessed via esthesiometry, showed statistically significant improvements in the central quadrant and across all quadrants on average. Central corneal sensitivity improved from 3.4 ± 1.9 cm pre-treatment to 5.0 ± 1.2 cm post-treatment (*p* = 0.005), while the mean sensitivity across all quadrants increased from 3.0 ± 1.7 cm pre-treatment to 4.7 ± 1.2 cm post-treatment (*p* = 0.002) (Table 3).

**Table 2:** Clinical parameters at baseline (V0) and after treatment (V1)

	V0	V1	*p* value*
BCVA (logMAR)Mean ± SD; Median (IQR)	0.17 ± 0.15; 0.13 (0.22)	0.10 ± 0.13; 0.05 (0.19)	0.075
Schirmer Test (mm/5 min) Mean ± SD; Median (IQR)	10.3 ± 4.7; 10.0 (7.5)	19.7 ± 12.1; 16.0 (19.5)	0.041
NIBUT (sec.) Mean ± SD; Median (IQR)	7.2 ± 3.9; 6.0 (4.6)	6.9 ± 3.3; 6.0 (3.5)	0.590
Oxford score Mean ± SD; Median (IQR)	1.7 ± 1.0; 1.0 (1.8)	0.8 ± 0.8; 1.0 (1.0)	0.008
OSDI score Mean ± SD; Median (IQR)	21.1 ± 12.9; 17.0 (15.8)	17.0 ± 10.0; 13.0 (11.3)	0.009

*Wilcoxon signed-rank test

*BCVA* best corrected visual acuity, *SD *standard deviation, *IQR* interquartile range, *NIBUT* non-invasive break-up time, *OSDI* ocular surface disease index

**Table 3:** Corneal sensitivity and sub-basal nerve plexus parameters before (V0) and after treatment (V1)

	V0	V1	*p* value*
CCS (cm)Mean ± SD; Median (IQR)	3.4 ± 1.9; 4.0 (1.5)	5.0 ± 1.2; 5.3 (0.0)	0.005
MCS (cm) Mean ± SD; Median (IQR)	3.0 ± 1.7; 3.0 (1.9)	4.7 ± 1.2; 5.1 (0.8)	0.002
SCS (cm) Mean ± SD; Median (IQR)	2.6. ± 1.9; 2.5 (2.8)	4.2 ± 1.9; 5.0 (2.1)	0.008
NCS (cm) Mean ± SD; Median (IQR)	2.6 ± 1.7; 3.0 (2.0)	4.4 ± 1.3; 4.5 (1.0)	0.014
ICS (cm) Mean ± SD; Median (IQR)	3.0 ± 1.8; 4.0 (2.0)	4.9 ± 1.3; 5.0 (1.8)	0.014
TCS (cm) Mean ± SD; Median (IQR)	3.4 ± 1.9; 4.0 (1.9)	5.1 ± 1.6; 6.0 (0.9)	0.006
CNFL (mm/mm²) Mean ± SD; Median (IQR)	11.0 ± 6.4; 11.5 (6.7)	14.7 ± 7.3; 15.7 (11.7)	0.006
CNFD (fibers/mm²) Mean ± SD; Median (IQR)	23.1 ± 13.2; 18.8 (10.9)	33.8 ± 11.9; 34.4 (6.7)	0.012
CNBD (branches/mm^2^) Mean ± SD; Median (IQR)	18.8. ± 14.1; 18.8 (10.9)	25.0 ± 14.1; 21.9 (21.9)	0.071

*Wilcoxon signed-rank test

*CCS* central corneal sensitivity, *SD* standard deviation, *IQR* interquartile range, *MCS* mean corneal sensitivity, *SCS* superior corneal sensitivity, *NCS *nasal corneal sensitivity, *ICS* inferior corneal sensitivity, *TCS *temporal corneal sensitivity, *CNFD* corneal nerve fiber density, *CNFL* corneal nerve fiber length, *CNBD* corneal nerve fiber total branch density

Non-invasive tear breakup time (NIBUT) did not show a significant change, with scores of 7.2 ± 3.9 s pre-treatment and 6.9 ± 3.3 s post-treatment (*p* = 0.590) (Table 2).

Ocular discomfort, measured via the Ocular Surface Disease Index (OSDI), improved significantly, with scores decreasing from 21.1 ± 12.9 pre-treatment to 17.0 ± 10.0 post-treatment (*p* = 0.009). Similarly, the Oxford score significantly improved, with values decreasing from 1.7 ± 1.0 pre-treatment to 0.8 ± 0.8 post-treatment (*p* = 0.008) (Table 2).

### In vivo confocal microscopy findings

**Fig. 1:**
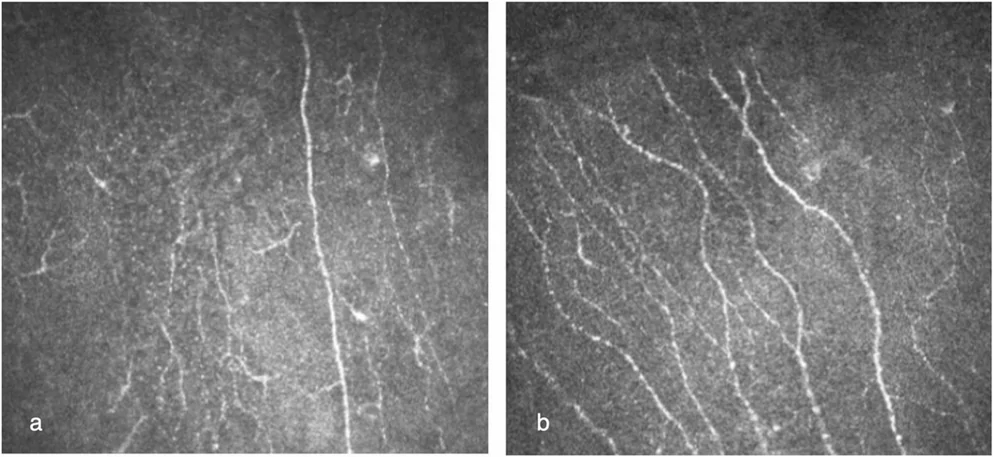
In vivo confocal microscopy images of the corneal sub-basal nerve plexus: (**a**) pre-treatment image showing reduced nerve fiber density and tortuosity. (**b**) Post-treatment image showing increased nerve fiber length and branching

IVCM analysis revealed significant improvements in corneal nerve parameters following treatment. CNFL improved significantly, increasing from 11.0 ± 6.4 mm/mm² pre-treatment to 14.7 ± 7.3 mm/mm² post-treatment (*p* = 0.006). Similarly, CNFD increased significantly from 23.1 ± 13.2 fibers/mm² pre-treatment to 33.8 ± 11.9 fibers/mm² post-treatment (*p* = 0.012). Although CNBD increased from 18.8 ± 14.1 branches/mm² pre-treatment to 25.0 ± 14.1 branches/mm² post-treatment, this change did not reach statistical significance (*p* = 0.071) (Table 3) (Fig. 1).

In the correlation analysis of structural microscopic measurements, CNFL (*p* < 0.01) and CNBD (*p* < 0.05) after ASED application were inversely associated with both baseline and post-treatment OSDI scores. Moreover, CNFD at the end of follow-up negatively correlated with baseline NIBUT (*p* < 0.05) (Table 4) (see Supplemental Digital Content 1, a heatmap illustrating the correlation matrix between clinical variables and IVCM metrics).

**Table 4:** Significant correlations between clinical, functional, and morphological parameters at baseline (V0) and post-treatment (V1). Only statistically significant associations (*p* < 0.05) are reported. Spearman’s rank correlation coefficients (r) are reported

Variable 1	Variable 2	Correlation (*r*)	*p* value*
CNFL V0	OSDI V1	-0.634	*p* < 0.05
CNFL V1	OSDI V0	-0.768	*p* < 0.01
CNFL V1	OSDI V1	-0.866	*p* < 0.01
CNBD V1	OSDI V0	-0.641	*p* < 0.05
CNBD V1	OSDI V1	-0.694	*p* < 0.05
CNFD V1	NIBUT V0	-0.658	*p* < 0.05
CCS V0	NIBUT V0	0.677	*p* < 0.05
CCS V0	NIBUT V1	0.698	*p* < 0.05
CCS V1	Oxford score V1	-0.721	*p* < 0.05
MCS V1	NIBUT V0	0.662	*p* < 0.05
MCS V1	NIBUT V1	0.719	*p* < 0.05

*CNFL* corneal nerve fiber length, *CNBD* corneal nerve fiber total branch density, *CNFD *corneal nerve fiber density, *CCS* central corneal sensitivity, *MCS* mean corneal sensitivity, *OSDI* ocular surface disease index, *NIBUT* non-invasive break-up time

Regarding corneal sensitivity, baseline central corneal sensitivity (CCS) and post-treatment mean corneal sensitivity (MCS) positively correlated with NIBUT at both baseline and post-treatment evaluations (*p* < 0.05). Additionally, post-treatment CCS showed an inverse correlation with the Oxford score measured at the end of follow-up (*p* < 0.05) (Table 4) (see Supplemental Digital Content 2, a heatmap illustrating the correlation matrix between clinical variables and corneal sensitivity).

No significant correlations were found between corneal sensitivity and IVCM parameters (see Supplemental Digital Content 3, a heatmap illustrating the correlation matrix between IVCM metrics and corneal sensitivity).

## Discussion

This prospective study showed that the use of 20% ASED in patients with PL and NK led to significant improvements in tear production, corneal staining, corneal sensitivity, and ocular symptoms. Additionally, IVCM analysis revealed significant increases in CNFL and CNFD suggesting structural recovery of the CSNP and highlighting the neurotrophic potential of this treatment.

ASED are widely used to manage ocular surface disorders, including severe dry eye disease, NK, and tear dysfunction in graft-versus-host disease. Treatment with 20–100% autologous serum, applied four to eight times daily, has shown improvements in symptoms such as dryness and discomfort. Clinical benefits have also been reported in corneal and conjunctival staining, Schirmer test, tear film break-up time, and corneal sensitivity [20–23]. Our findings are consistent with this evidence and further support the therapeutic potential of ASED. These effects are likely mediated by its bioactive components—including albumin, vitamin A, TGF-β, and EGF—which promote epithelial health and tear film stability [11]. Moreover, the concentration of these bioactive components may vary not only depending on the final dilution but also on preparation parameters such as clotting time, centrifugation, and diluent, which modulate the biochemical and epitheliotrophic profile of ASED [19, 23]. 

In our cohort, one patient discontinued treatment after two weeks due to ocular discomfort, though no signs of infection were observed. Autologous serum contains bactericidal and bacteriostatic molecules, such as lysozyme, lactoferrin, and immunoglobulins, which reduce the risk of contamination [10]. It is generally well tolerated when prepared and stored under sterile conditions [24]. However, elevated levels of certain cytokines—such as TGF-β, interleukin-17, and interferon— may exert antiproliferative or proinflammatory effects, potentially contributing to adverse responses [23, 25]. This highlights the need for further investigation into the individual variability of ASED composition and its tolerability [26]. 

Managing PL in the context of NK is particularly challenging due to their synergistic impact on ocular surface integrity, reflecting a combined pathophysiological condition in which neurotrophic impairment and chronic exposure-related surface damage coexist and interact. In patients with neurosurgical history, trigeminal pathway impairment may occur through tumor compression, surgical manipulation within the cerebellopontine angle and posterior fossa, or involvement of central trigeminal pathways, leading to reduced corneal trophic support [5, 6]. At the same time, long-standing lagophthalmos acts as an independent pathogenic factor, as chronic ocular surface exposure and persistent inflammation are recognized contributors to NK due to their direct effects on corneal nerves and epithelial trophism [5]. Together, these conditions may lead to corneal ulcers, stromal scarring, and perforation, often requiring surgical intervention to restore corneal transparency and visual function [27, 28]. However, the neurotrophic component frequently limits the feasibility of keratoplasty, prompting the use of alternative strategies such as conjunctival flaps, amniotic membrane transplantation, or tarsorrhaphy. Despite their effectiveness in preserving structural integrity, these interventions can result in suboptimal functional and aesthetic outcomes [29]. 

A retrospective study by Kim et al. evaluated autologous serum gel in 15 patients with refractory neurotrophic epithelial defects associated with lagophthalmos [30]. The treatment led to significant reductions in epithelial defect size, improved visual acuity, and relief of ocular discomfort in cases unresponsive to conventional therapies. However, while corneal healing occurred in most patients, the study did not assess corneal sensitivity or nerve regeneration using advanced imaging such as IVCM.

Our study showed that treatment with 20% ASED led to significant improvements in corneal nerve parameters observed by IVCM. Specifically, CNFL and CNFD increased after two months of treatment, suggesting structural nerve regeneration. Specifically, we observed inverse correlations between CNFL and CNBD with both baseline and post-treatment OSDI scores, suggesting that greater corneal nerve integrity may help mitigate subjective symptom severity. Additionally, the negative correlation between CNFD at the end of follow-up and baseline NIBUT supports the idea that higher nerve fiber density is associated with better tear film stability. These findings align with previous observations by Tepelus et al., who reported a significant association between reduced nerve density and increased OSDI scores in both non-Sjögren’s and Sjögren’s dry eye patients, reinforcing the value of OSDI as a reliable tool for assessing dry eye symptoms in relation to sub-basal nerve plexus alterations [31]. 

Corneal sensitivity, assessed by esthesiometry, also improved significantly across all quadrants, indicating that morphological recovery was accompanied by functional restoration. Moreover, the observed correlations between corneal sensitivity, NIBUT, and the Oxford score suggest a functional interplay between corneal innervation, tear film stability, and epithelial integrity. Our findings are consistent with those of Aggarwal et al., who evaluated 16 patients with neuropathic corneal pain and reported improvements in total nerve length and density following ASED therapy [13]. In their study, reductions in nerve morphological abnormalities—such as beading and micro-neuromas—were associated with decreased pain severity, supporting the potential for functional nerve recovery. In a previous study on 16 patients with severe photoallodynia, the same group reported comparable benefits: ASED therapy 8 times daily over 3.6 months led to significant nerve regeneration on IVCM and a marked reduction in light sensitivity [18]. 

Similarly, Abazari et al. evaluated the effects of ASED in four patients with severe NK secondary to herpes zoster ophthalmicus. Their findings demonstrated significant corneal nerve regeneration and restoration of corneal sensation, as measured by IVCM and esthesiometry, respectively. Total nerve density increased to a mean of 10,085.88 ± 2542.74 μm/mm² at follow-up, while corneal sensitivity improved from 0 cm to 3.50 ± 1.30 cm [32]. 

These effects are likely mediated by the bioactive components of autologous serum, including high concentrations of neurotrophic and epithelial growth factors such as nerve growth factor (NGF), brain-derived neurotrophic factor (BDNF), and glial-derived neurotrophic factor (GDNF) [22, 33]. 

Among these, NGF plays a key role in nerve regeneration by promoting neuronal sprouting and facilitating functional recovery of injured neurons. In both in vitro and in vivo models, NGF supports the differentiation and growth of sensory and sympathetic neurons. Its synthesis in adult tissues correlates with increased innervation density, axonal sprouting, dendritic arborization, and the release of neuropeptides and neurotransmitters [34, 35]. Autologous serum also contains other critical factors, including EGF, insulin-like growth factor-1, TGF-β, and substance P (SP) [11]. SP plays a crucial role in neuro-immune crosstalk, enhancing corneal epithelial wound healing and promoting axonal outgrowth. As a key mediator of neuroinflammation, SP contributes to the resolution of injury-induced inflammation while fostering an environment conducive to nerve regeneration and epithelial integrity [36]. These combined effects underscore the importance of restoring epithelial-neural interactions to re-establish corneal homeostasis. Interestingly, Abazari et al. reported that three out of four patients developed stromal keratitis shortly after nerve recovery, highlighting the complex relationship between regeneration and inflammation—an aspect that should be carefully considered in neuroregenerative therapies [32]. 

Notably, despite an upward trend in CNBD, the change was not statistically significant. This may indicate that branch formation—characterized by Y- and H-shaped patterns on IVCM [3]—requires longer treatment or adjunctive therapies.

This study has some limitations. Most notably, the absence of a control group treated with artificial tears limits the ability to draw definitive conclusions regarding the neuro-regenerative role of ASED. However, this limitation is primarily due to the rarity and heterogeneity of patients presenting with combined trigeminal and facial nerve dysfunction, which made it impractical to assemble a well-matched control cohort. Moreover, all enrolled patients were already receiving long-term treatment with preservative-free artificial tears prior to inclusion, without achieving adequate clinical control of ocular surface disease. Therefore, continuing standard therapy alone was considered suboptimal from a clinical perspective. Further controlled studies incorporating appropriate control groups and rescue therapies are warranted. Nevertheless, the observational data collected provide valuable preliminary insights into the potential role of ASED in managing this complex and underserved patient population. The small sample size, while sufficient to demonstrate statistically significant changes, limits generalizability and does not allow for subgroup analyses. In addition, the relatively short follow-up period may not fully reflect long-term outcomes or delayed complications of ASED therapy. Although IVCM provided valuable structural information, the inclusion of complementary tools, such as molecular biomarkers or functional imaging, could further clarify the mechanisms underlying nerve recovery [37]. The restricted field of view and the inability to image the exact same corneal location over time, inherent limitations of IVCM, may introduce variability in image selection; however, this was mitigated by using consistent scanning protocols and selecting representative images at the same depth from the central cornea. Additionally, the correlations observed at the end of the follow-up may require further investigation to be fully elucidated. Future studies with larger cohorts, control groups, and extended follow-up will help validate and expand on these results. The strengths of the study include its prospective design and the strict inclusion and exclusion criteria.

In conclusion, this study highlights the potential of 20% ASED to promote corneal nerve regeneration, enhance corneal sensitivity, and restore ocular surface health in patients with PL and NK. These findings support the neurotrophic and regenerative properties of ASED as a promising therapeutic option for complex ocular surface diseases. Larger, long-term studies are warranted to confirm these results and optimize treatment protocols. The integration of advanced imaging tools, such as IVCM, may further support personalized approaches and improve outcomes in neurotrophic ocular conditions.

## Supplementary Information

Below is the link to the electronic supplementary material.

Supplementary Material 1

Supplementary Material 2
