# Supplementary material for: Corneal nerve regeneration and ocular surface restoration following treatment with autologous serum eye drops in patients with paralytic lagophthalmos and neurotrophic keratitis
Source: Graefes Arch Clin Exp Ophthalmol. 2026 Jul 17;264(11):3429–37. doi: 10.1007/s00417-026-07388-w (PMC13736426; doi:10.1007/s00417-026-07388-w)

Corneal Nerve Regeneration and Ocular Surface Restoration Following Treatment with Autologous Serum Eye Drops in Patients with Paralytic Lagophthalmos and Neurotrophic Keratitis.

Graefe's Archive for Clinical and Experimental Ophthalmology

Luca Lucchino[1], MD, Elvia Mastrogiuseppe[1]*, MD, Francesca Giovannetti[1], MD, Mattia D'Andrea[1], MD, Giulia Mecarelli[1], MD, Alice Bruscolini[1], MD, PhD, Alessandro Lambiase[1], MD, PhD, Marco Marenco[1], MD, PhD

[1] Department of Sense Organs, Sapienza University of Rome, Rome, Italy

*Corresponding Author: Elvia Mastrogiuseppe, MD

Department of Sense Organs, Sapienza – University of Rome, Viale del Policlinico 155, 00161 Rome, Italy

Email elvia.mastrogiuseppe@uniroma1.it; Tel +39 0649975389

Supplementary Fig 1 Heatmap displaying the associations between clinical variables and IVCM metrics. Red cells indicate negative correlations, whereas blue cells denote positive ones. Each cell contains the corresponding Spearman correlation coefficient. Statistical significance is marked as follows: * $p < 0.05$; ** $p < 0.01$; *** $p < 0.001$.

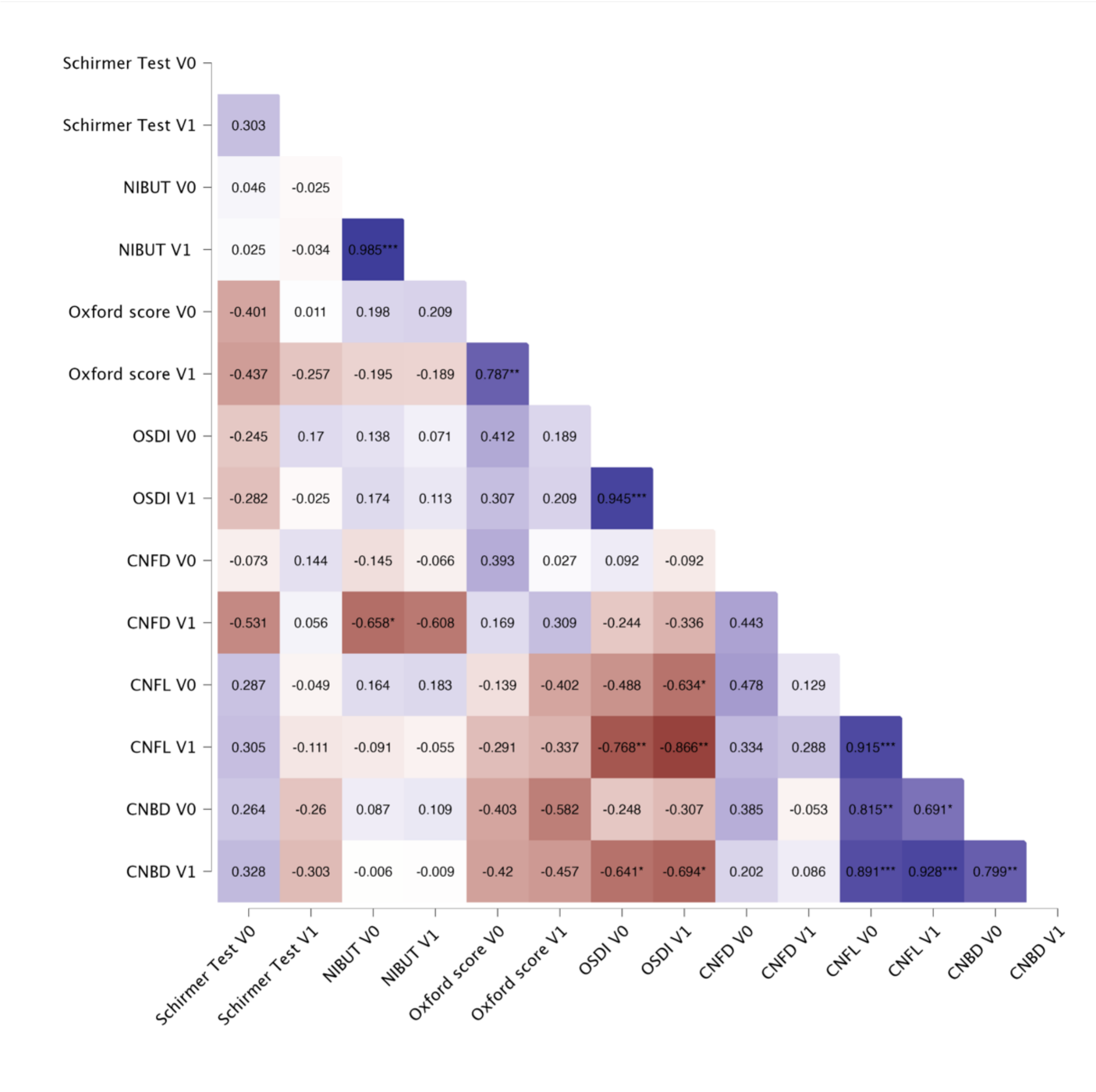

Supplementary Fig 2 Heatmap displaying the associations between clinical variables and corneal sensitivity. Red cells indicate negative correlations, whereas blue cells denote positive ones. Each cell contains the corresponding Spearman correlation coefficient. Statistical significance is marked as follows: * p < 0.05; ** p < 0.01; *** p < 0.001.

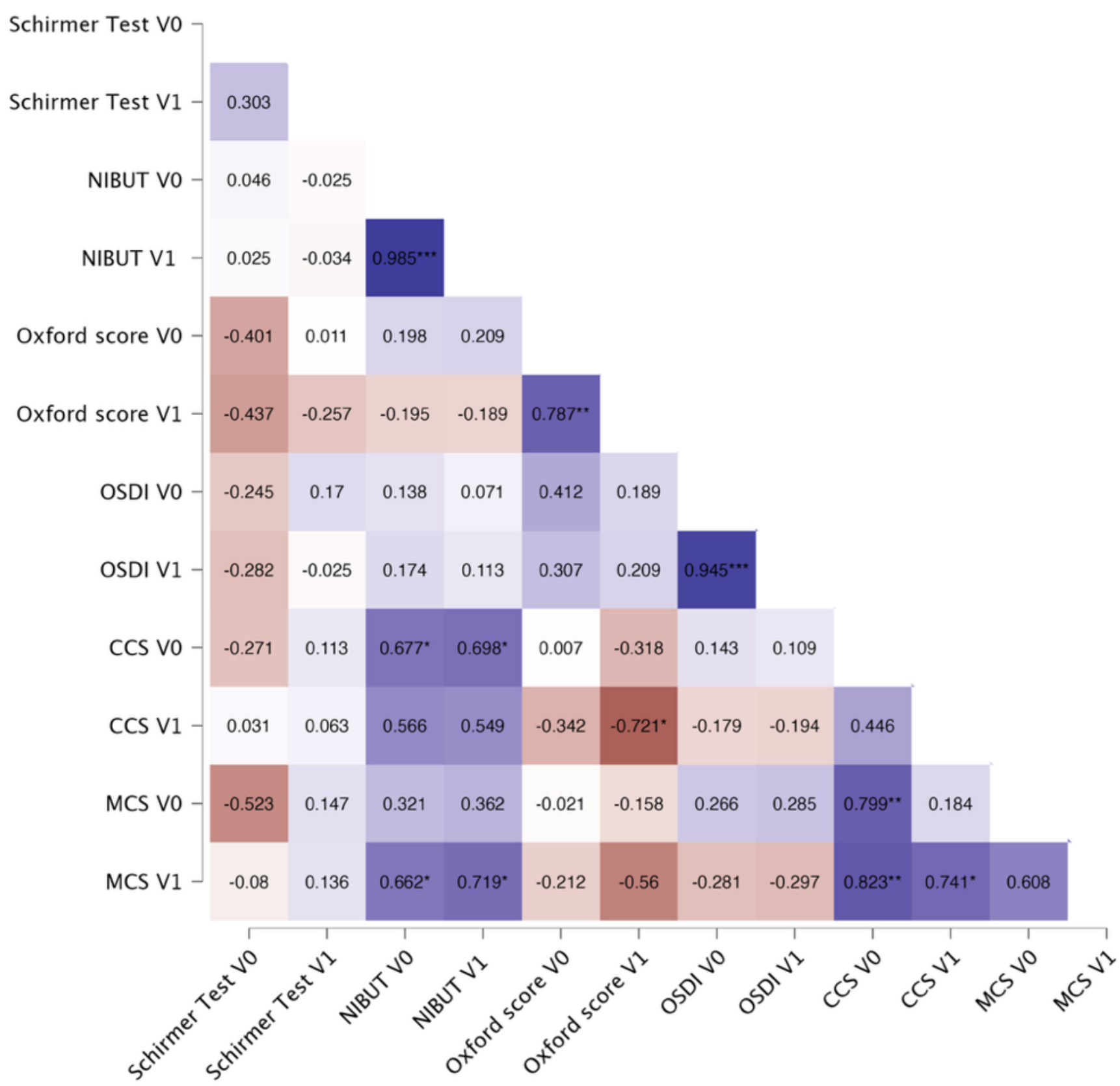

Supplementary Fig 3 Heatmap displaying the associations between IVCM metrics and corneal sensitivity. Red cells indicate negative correlations, whereas blue cells denote positive ones. Each cell contains the corresponding Spearman correlation coefficient. Statistical significance is marked as follows: * $p < 0.05$; ** $p < 0.01$; *** $p < 0.001$.

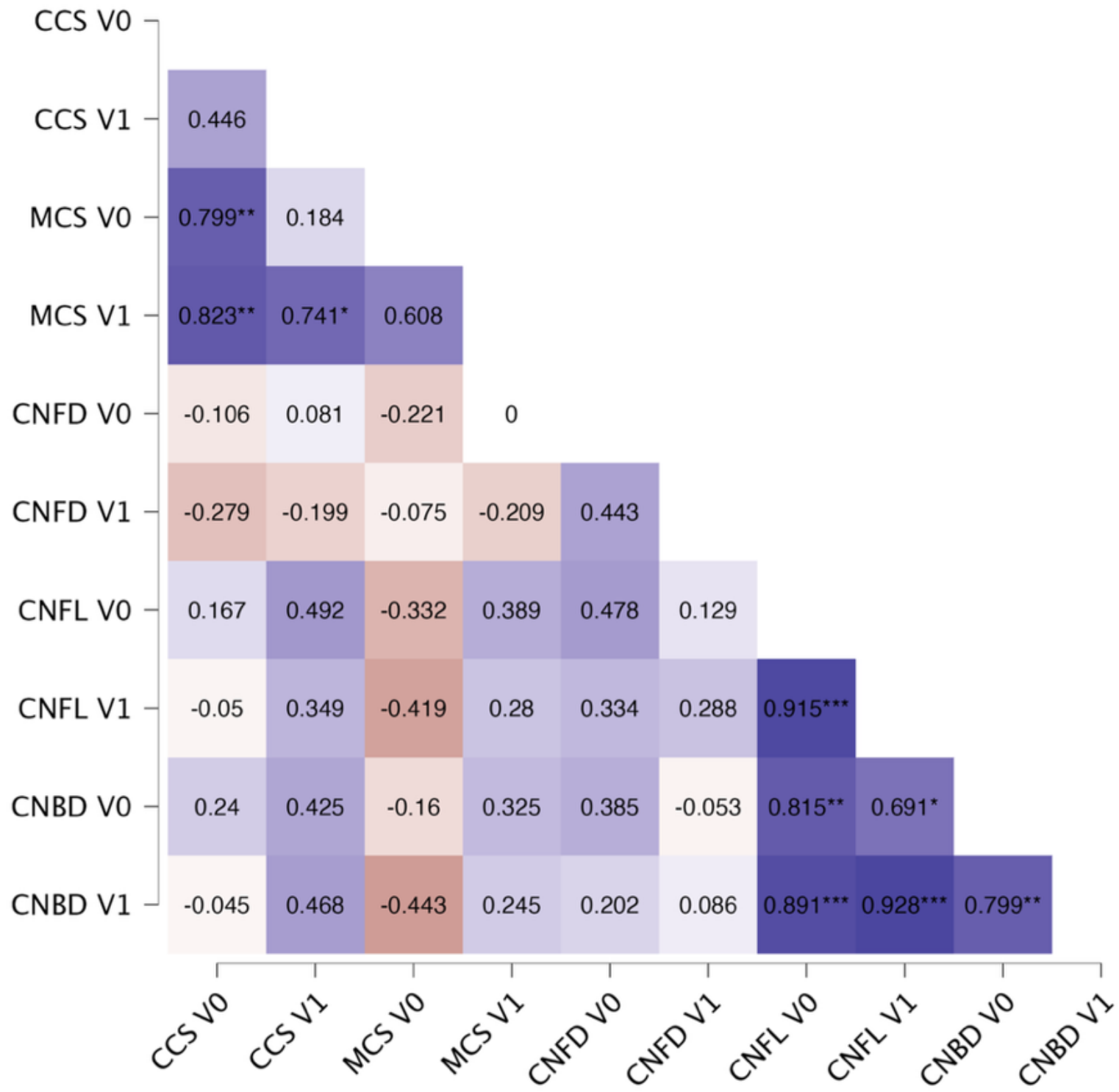

Supplement: Supplementary file 1 [file 417_2026_7388_MOESM1_ESM.pdf]
