# Supplementary material for: Corneal nerve regeneration and ocular surface restoration following treatment with autologous serum eye drops in patients with paralytic lagophthalmos and neurotrophic keratitis
Source: Graefes Arch Clin Exp Ophthalmol. 2026 Jul 17;264(11):3429–37. doi: 10.1007/s00417-026-07388-w (PMC13736426; doi:10.1007/s00417-026-07388-w)
Supplement: Supplementary file 2 [file 417_2026_7388_MOESM2_ESM.docx]

**Corneal Nerve Regeneration and Ocular Surface Restoration Following Treatment with Autologous Serum Eye Drops in Patients with Paralytic Lagophthalmos and Neurotrophic Keratitis.**

Graefe's Archive for Clinical and Experimental Ophthalmology

Luca Lucchino^1^, MD, Elvia Mastrogiuseppe^1*^, MD, Francesca Giovannetti^1^, MD, Mattia D’Andrea^1^, MD, Giulia Mecarelli^1^, MD, Alice Bruscolini^1^, MD, PhD, Alessandro Lambiase^1^, MD, PhD, Marco Marenco^1^, MD, PhD

^1^ Department of Sense Organs, Sapienza University of Rome, Rome, Italy

^*^Corresponding Author: Elvia Mastrogiuseppe, MD

Department of Sense Organs, Sapienza – University of Rome, Viale del Policlinico 155, 00161 Rome, Italy

Email elvia.mastrogiuseppe@uniroma1.it; Tel +39 0649975389

**Supplementary Fig. 4. Slit-lamp images of the ocular surface before and after treatment with 20% autologous serum eye drops (ASED).** (A,B) Baseline slit-lamp photographs under white light (A) and cobalt blue illumination with fluorescein staining (B) showing superficial punctate keratopathy consistent with stage 1 NK in a patient with paralytic lagophthalmos.

(C,D) Slit-lamp images obtained after 2 months of treatment with 20% ASED under white light (C) and cobalt blue illumination (D), demonstrating improvement of the ocular surface with reduction of corneal staining and epithelial irregularities.
